# *Lactiplantibacillus plantarum* Lac16 Attenuates Enterohemorrhagic *Escherichia coli* O157:H7 Infection by Inhibiting Virulence Traits and Improving Intestinal Epithelial Barrier Function

**DOI:** 10.3390/cells12101438

**Published:** 2023-05-21

**Authors:** Baikui Wang, Yuanhao Zhou, Qi Wang, Shujie Xu, Fei Wang, Min Yue, Zhonghua Zeng, Weifen Li

**Affiliations:** 1 Key Laboratory of Molecular Animal Nutrition of the Ministry of Education, Institute of Animal Nutrition and Feed Sciences, Zhejiang University College of Animal Sciences, Hangzhou 310058, China; wangbaikui@zju.edu.cn (B.W.); zyh17767072477@163.com (Y.Z.); 12117010@zju.edu.cn (Q.W.); 22017075@zju.edu.cn (S.X.); 22017007@zju.edu.cn (F.W.); 2 Department of Veterinary Medicine, Institute of Preventive Veterinary Sciences, College of Animal Sciences, Zhejiang University, Hangzhou 310012, China; myue@zju.edu.cn; 3 Nanjing Kangyou Biotechnology Co., Ltd., Nanjing 211316, China; 21717008@zju.edu.cn

**Keywords:** *Lactiplantibacillus plantarum*, enterohemorrhagic *E. coli* O157:H7, epithelial damage, barrier dysfunction, Wnt/β-catenin pathway

## Abstract

Large-scale use of antimicrobials in agriculture and medicine contributes to antibiotic residues in raw foods, the spread of antimicrobial resistance (AMR) and drug pollution, which seriously threatens human health and imposes significant economic burdens on society, suggesting the need for novel therapeutic options that prevent or control zoonoses. In this study, four probiotics were selected to assess their capability to alleviate pathogen-induced damage. Results showed that a simulated gastrointestinal juice and bile tolerated *L. plantarum* Lac16 with high lactic acid secretion can significantly inhibit the growth of multiple zoonotic pathogens. Lac16 also significantly inhibited the biofilm formation and mRNA expression of virulence traits (genes related to virulence, toxins, flagella biogenesis and motility, antibiotic resistance, biofilm formation and AI-2 quorum sensing) of enterohemorrhagic *E. coli* O157:H7 (EHEC). Furthermore, Lac16 and Lac26 significantly protected *C. elegans* against zoonotic pathogen-induced (EHEC, *S. typhimurium*, *C. perfringens*) deaths. Moreover, Lac16 significantly promoted epithelial repair and ameliorated lipopolysaccharide (LPS)-induced intestinal epithelial apoptosis and barrier dysfunction by activating the Wnt/β-catenin signaling pathway, and markedly reduced LPS-induced inflammatory responses by inhibiting the TLR4/MyD88 signaling pathway. The present results indicate that Lac16 attenuates enterohemorrhagic *E. coli* infection-induced damage by inhibiting key virulence traits of *E. coli*, promoting epithelial repair and improving intestinal epithelial barrier function, which may be mediated by the activated Wnt/β-catenin signaling pathway and the inhibited TLR4/MyD88 signaling pathway of the intestinal epithelium.

## 1. Introduction

Although there are many benefits to interacting with livestock (pigs, cattle, sheep, goats), poultry and pets, it is also vital to be aware that these domesticated animals usually carry lots of harmful germs (bacteria, fungi, viruses, parasites, etc.) that can cause a variety of illnesses, ranging from minor skin infections to serious gastrointestinal infectious diseases [1,2]. Zoonotic diseases induced by pathogens (e.g., *E. coli* species, *Salmonella* species, *Staphylococcus aureus*, *Listeria monocytogenes*, *Clostridium perfringens*, *Candida* species, etc.) that are shared between vertebrate animals and humans seriously threaten the health of animals and humans by causing many foodborne illnesses, ranging from mild to serious, and even death [1,3,4]. This is one of the greatest challenges to global health security. Meanwhile, the use of antimicrobials in animal husbandry and medicine contributes to antimicrobial residues in raw food animal products (meat, eggs, dairy), the spread of antimicrobial resistance (AMR) and drug environmental pollution [5,6,7,8]. Many studies have reported that zoonotic disease recurrence [9], antimicrobial resistance [10] and foodborne antimicrobial residues [11] emerging after antibiotic treatments impose a huge economic burden on the global health system and inspires an interest in seeking novel therapeutic strategies, as alternatives to antimicrobials, that prevent or control zoonosis outbreaks [10,12].

The intestinal epithelial barrier plays a crucial role in limiting interactions between luminal contents (e.g., intestinal microbes, water, nutrients, ions, waste products) and the underlying immune system, preventing the invasion of zoonotic pathogens and maintaining the integrity of the intestinal epithelium and intestinal homeostasis [13,14,15]. The signals derived from invading pathogens or commensal microbes can be picked up by intestinal epithelial cells and then be relayed to immune cells in the lamina propria to modulate intestinal epithelial barrier functions or host defense [15,16,17]. It is reported that zoonotic pathogens induce toxicity or damage to host tissues or cells by biofilm formation and overgrowth regulated by quorum-sensing systems, releasing toxins, adhering to and invading intestinal epithelial cells, translocating to host organs, or provoking host excessive immune response [18,19,20,21,22], which provides potential therapeutic targets to prevent or control zoonotic diseases.

As an attractive “Generally Recognized as Safe (GRAS)” alternative to antimicrobials, probiotics without residues in raw food products exert beneficial effects on a host’s overall health by producing organic acids and antimicrobial compounds; restoring gut microbiota; interacting with resident microbiota or the host; inhibiting the overgrowth, adhesion and invasion of zoonotic pathogens; enhancing gastrointestinal epithelial barrier function; improving digestive capacity and modulating mucosal immune responses [12,23,24,25]. These provide potential therapeutic options to prevent or control infection of zoonotic pathogens. In the present study, four probiotics were selected to assess their capability to alleviate zoonotic pathogen-induced toxicity to *Caenorhabditis elegans* and LPS exposure-induced intestinal epithelial damage and the underlying mechanisms.

## 2. Materials and Methods

### 2.1. Bacteria

Four lactic acid bacteria isolated and purified from fermented vegetables were cultured in de Man-Rogosa-Sharpe (MRS) broth or agar plates (Hopebio, Qingdao, China) at 37 °C overnight under aerobic conditions. *Bacillus amyloliquefaciens* SC06 (BaSC06, CCTCC M2012280), enterohemorrhagic *Escherichia coli* O157:H7 (EHEC, ATCC43895), enterotoxigenic *Escherichia coli* (K88, K99, F18), *Escherichia coli* OP50, *Salmonella typhimurium* SL1344 (ST), *Salmonella enteritidis* (SE) and *Staphylococcus aureus* (SA) were cultured in Luria Broth (LB) or agar plates at 37 °C overnight under aerobic conditions, separately. *Clostridium perfringens* type A (Cp) was incubated in Reinforced clostridium medium (RCM) broth (Hopebio, Qingdao, China) at 37 °C under anaerobic conditions overnight. *Listeria monocytogenes* (LM) was incubated in Brain heart infusion (BHI) broth (Huankai Microbial, Guangzhou, China) at 37 °C under anaerobic conditions overnight. *Candida albicans* (CA) was incubated in Yeast extract peptone dextrose (YPD) broth (Huankai Microbial, Guangzhou, China) at 37 °C under anaerobic conditions overnight. After being centrifugated at 4000× *g* for 15 min at 4 °C, bacteria pellets were washed three times with sterile phosphate-buffered saline (PBS, pH 7.2) and then the final concentration was constantly checked by the spreading plate method. After being centrifugated at 4000× *g* for 15 min at 4 °C, the fermented supernatant of Lac16 was collected and then filtered through a 0.22 μm membrane (Merck Millipore, Burlington, MA, USA) and kept at 4 °C for further use.

### 2.2. Antimicrobial Activity

For the diffusion method, 45–50 °C agar medium (LB, YPD, BHI) containing 0.25% (*v*/*v*) different zoonotic pathogens at logarithmic growth phase were poured into 10 cm plates (Corning, NY, USA). Then, 200 μL of the sterile supernatant of the overnight fermented lactic acid bacteria was added into 8 mm agar wells created by punching the above-mentioned 10 cm agar plates. After overnight incubation at 37 °C, the antimicrobial activity was investigated as the zone of growth inhibition around the 8 mm agar wells.

For viability assays, the sterile supernatant of the overnight fermented lactic acid bacteria or 100 μg/mL gentamicin containing 0.25% (*v*/*v*) different zoonotic pathogens at logarithmic growth phase were diluted by a 10-fold serial dilution method and then plated onto BHI agar at 37 °C overnight to determine viability.

### 2.3. Aggregation Assay

An aggregation assay was conducted according to the method reported in a previous study [26]. Briefly, the final concentration of pathogens and probiotics were adjusted to 2 × 10^8^ CFU/mL. For auto-aggregation assay, the resuspended probiotics (5 mL) were added into sterile tubes separately and then placed at 37 °C without agitation. Then, an aliquot of 150 μL upper suspension was taken at 3 h intervals to measure the absorbance of OD_600nm_ using a SpectraMax M5 reader (Molecular Devices, Sunnyvale, CA, USA). Finally, the auto-aggregation rate was calculated by the equation: auto-aggregation rate (%) = (A0 − An)/T0 × 100%, where A0 is the OD_600nm_ value of upper suspension at 0h, and An is the OD_600nm_ value at different time points.

For the co-aggregation assay, equal volumes (1.5 mL) of lactic acid bacteria and pathogens cultures at logarithmic growth phase were mixed completely and placed at 37 °C without agitation. Then, an aliquot of 150 μL upper suspension was taken at 0 h and 4 h to measure the absorbance of OD_600nm_ using a SpectraMax M5 reader. Finally, the co-aggregation rate was calculated by the equation: co-aggregation rate (%) = [(A_pro_ + A_pat_) − 2 × A_mix_]/(A_pro_ + A_pat_) × 100%, where A_pro_ and A_pat_ are the OD_600nm_ value of lactic acid bacteria and pathogen cultures at 0 h, and A_mix_ is the OD_600nm_ value of the mixed resuspensions at 4 h.

### 2.4. Simulated Gastrointestinal Juices Resistance Assay

For the viable counts assay, lactic acid bacteria at logarithmic growth phase was added into 5 mL (1% *v*/*v*) of simulated gastric juices (Phygene, Fuzhou, China) with different pH values (2.5, 3.0, 4.0), and simulated intestinal juice (Phygene, Fuzhou, China) with different pH values (8.0), separately. The mixtures were incubated at 37 °C for 3 h for the evaluation of tolerance in gastric juices or for 24 h for the evaluation of tolerance in simulated intestinal juices. Finally, total viable counts were investigated at 3 h or 24 h for the evaluation of simulated gastrointestinal juices resistance by the spreading plate method on MRS agar. The survival rate was calculated by the equation: survival rate (%) = log(CFU_tn_)/log(CFU_t0_) × 100%, where CFU_tn_ is the total viable counts of lactic acid bacteria after treatment with simulated gastrointestinal juices, and CFU_t0_ is the total viable counts of lactic acid bacteria before treatment with simulated gastrointestinal juices.

For the growth curve assay, lactic acid bacteria at logarithmic growth phase was added into 20 mL (1% *v*/*v*) of simulated gastric juices with different pH values (2.5, 3.0, 4.0), and simulated intestinal juice with different pH values (8.0), separately. The mixtures were then plated into 96-well plates with 5 wells per group; then, the growth curve was measured using the Bioscreen C^®^ platform (OY Growth Curves Ab Ltd., Turku, Finland), and the absorbance of OD_600nm_ was measured every 10 min intervals for 73 h.

### 2.5. Bile Tolerance Assay

The bile tolerance assay was performed according to the method reported in a previous study [27]. The lactic acid bacteria at logarithmic growth phase were added into fresh MRS broth (1% *v*/*v*) containing different (*wt*/*v*, 0, 0.1%, 0.2%, 0.3%, 0.4%) porcine bile (SinoReagent, Shanghai, China) and then plated into 96-well plates with 5 wells per group; then, the growth curve was measured using the Bioscreen C^®^ platform, and the absorbance of OD_620nm_ was measured every 10 min intervals for 73 h. The incubated time required for the OD_620nm_ to increase by 0.3 units was calculated for the porcine bile-added culture of each strain and the difference in OD_620nm_ increase was recognized as the growth delay expressed in hours.

### 2.6. Lactic Acid Production

The lactic acid bacteria at logarithmic growth phase was added into 100 mL (1% *v*/*v*) fresh MRS broth and cultured at 37 °C, and 2 mL of the lactic acid bacteria cultures was taken at 6 h intervals for 96 h to measure the absorbance of OD_600nm_ using a SpectraMax M5 reader, the pH values (Thermo Fisher Scientific, Waltham, MA, USA) and the lactic acid levels by Lactic Acid assay kit (Nanjing Jiancheng Biology Engineering Institute, Nanjing, China).

### 2.7. Biofilm Formation

The final concentration of EHEC was adjusted to 2 × 10^6^ CFU/mL using the sterile supernatant of the fermented lactic acid bacteria (containing 20 g/L dextrose) or fresh MRS broth. The 200 μL resuspends were then added into 96-well plates with 7 wells per group and incubated at 37 °C for 5–7 days. After washing with sterile PBS, the incubated wells were stained with 200 μL 0.4% crystal violet for 30 min at room temperature and then washed with sterile PBS again. Finally, the cells were added 200 μL 75% ethanol to dissolve the stained crystal violet and the absorbance at 590 nm was measured by SpectraMax M5 reader.

### 2.8. Gene Expression of EHEC

The final concentration of EHEC was adjusted to 2 × 10^8^ CFU/mL using the sterile supernatant of the fermented lactic acid bacteria (containing 20 g/L dextrose) or fresh MRS broth. After being cultured (37 °C, 180 rpm/min) for 4 h, the EHEC pellets were collected by centrifugation (4 °C, 5000× *g*, 15 min) and washed three times with sterile PBS. The collected EHEC pellets were added to 1 mL RNAiso Plus (TAKARA, Dalian, China) for total RNA extraction; the reverse-transcription was conducted using the PrimeScript II 1st Strand cDNA Synthesis Kit (TAKARA, Dalian, China) according to the manufacturer’s instructions. The quantitative real-time PCR (qPCR) was then investigated using a StepOne real-time PCR system (Applied Biosystems) using SYBR PremixEx TaqII (TAKARA, Dalian, China). The qPCR primer sets for EHEC are list in Table S1. Fold changes were calculated after normalizing to two housekeeping genes (rpoA and 16S rRNA) using the 2^−ΔΔCt^ method [28].

### 2.9. Caenorhabditis Elegans Experiment

*C. elegans* N2 (Bristol) were routinely maintained on Nematode Growth Media (NGM) and fed with *E. coli* OP50 according to the description in WormBook [29]. To prepare L1-synchronized worms, eggs purified by dissolving gravid worms in NaOH-buffered bleach were isolated and then placed into 10 mL of M9 buffer for hatching overnight. Synchronized L1 worms were then incubated at 25 °C until the L4 larval stage on the NGM agar containing with *E. coli* OP50.

For a lifespan assay, the final concentration of probiotics at logarithmic growth phase were adjusted to 2 × 10^8~9^ CFU/mL using M9 medium. An amount of 200 μL of bacteria suspension was then poured onto NGM in 24-well plates (Corning, NY, USA), separately, following being dried at 22 °C for 4 h. L4-stage worms were transferred to lawns of bacteria (lactic acid bacteria, BaSC06, *E. coli* OP50) grown on NGM plates (30 worms/plate) with 3 plates per group. Worms were transferred to fresh bacterial lawns grown on NGM plates every 3 days and worm survival was monitored at 24 h intervals for fifteen days.

A *C. elegans* infection assay was performed according to a previous study [30]. Briefly, the final concentration of bacteria (lactic acid bacteria, BaSC06, pathogens) were adjusted to 2 × 10^8^ CFU/mL using M9 medium. An amount of 200 μL of probiotics suspension was poured onto NGM in 24-well plates, separately, and dried at 22 °C for 4 h. L4-stage worms were transferred to lawns of bacteria (lactic acid bacteria, BaSC06, *E. coli* OP50) grown on NGM plates (30 worms/plate) with 3 plates per group for colonization for 1 day. Then, the treated worms were washed and transferred to lawns of pathogens (EHEC, ST, Cp) grown on NGM plates for infection for another day. Finally, the infected worms were washed and transferred to lawns of *E. coli* OP50 grown on NGM plates (Day 0). Worm survival was monitored at 24 h intervals for fifteen days.

### 2.10. IPEC-J2 Cell Culture

The intestinal porcine enterocyte cell line (IPEC-J2) was cultured in DMEM/F12 full medium [DMEM/F12 (Gibco, Waltham, MA, USA) supplemented with 10% fetal bovine serum (Gibco, MA, USA), 100 U/mL penicillin and 100 μg/mL streptomycin (Sigma-Aldrich, St. Louis, MO, USA)] at 37 °C in a cell culture incubator with 90% humidity and 5% CO_2_.

### 2.11. Wound-Healing Assay

IPEC-J2 cells (2 × 10^6^ cells/well) seeded into 12-well plates (Corning, NY, USA) were cultured in DMEM/F12 full medium for three days. The IPEC-J2 cell monolayers were then scratched using a sterile 200 μL pipette tip and washed with PBS for 4–5 times to remove the cell debris. The scratched cells were treated with sterile PBS, Lac16 (MOI = 100), BaSC06 (MOI = 100, positive control), or lipopolysaccharide (LPS, 40 µg/mL). The dishes were placed at 37 °C in a 5% CO_2_ air atmosphere and the images of scratched cells were captured with a Leica DMI3000B (Leica, Wetzlar, Germany) at 0 h, 6 h, 12 h, 24 h, 36 h and 48 h. The wound closure of IPEC-J2 cells were measured by ImageJ software version 1.53 (National Institutes of Health, Bethesda, MD, USA).

### 2.12. CCK-8 Assay and Cell Apoptosis Detection

IPEC-J2 cells (1 × 10^4^ cells/well) seeded into 96-well plates were preincubated with sterile PBS, Lac16 (MOI = 100) or BaSC06 (MOI = 100, positive control) with 6 wells per group for 12 h. After washing with sterile PBS for 3 times to remove the bacteria, the treated IPEC-J2 cells were then treated with LPS (40 µg/mL) for another 12 h. Then the cell viability of the treated cells was investigated using a Cell Counting Kit-8 (Beyotime, Shanghai, China) according to the manufacturer’s instructions. Cell apoptosis was measured using an Annexin V-FITC Apoptosis Detection Kit (Beyotime, Shanghai, China) according to the manufacturer’s instructions and the results were analyzed by flow cytometry (Beckman Coulter, Fullerton, CA, USA).

### 2.13. LDH and NO Release Analysis, qPCR Assay

The IPEC-J2 cell monolayers seeded into 12-well plates were preincubated with sterile PBS, Lac16 (MOI = 100) or BaSC06 (MOI = 100, positive control) with 3 wells per group for 12 h. After washing with sterile PBS for 3 times to remove bacteria, the treated IPEC-J2 cells were then treated with LPS (40 µg/mL) for another 12 h. After capturing using Leica DMIRB, the supernatant of the treated cells was then collected to analysis the release of LDH and NO from cells using LDH kits (Beyotime, Shanghai, China) and NO kits (Beyotime, Shanghai, China) according to the manufacturer’s instructions. After washing with sterile PBS 3 times, the cells were collected by RNAiso Plus for total RNA extraction and qPCR. The primers of IPEC-J2 cells are shown in Table S2. Fold changes were calculated after normalizing to two housekeeping genes (β-actin and GAPDH) using the 2^−ΔΔCt^ method.

### 2.14. Western Blotting Analysis

After washing with PBS 3 times, the treated IPEC-J2 cells (as described in qPCR assay) were lysed using the RIPA Lysis Buffer (Beyotime, Shanghai, China) containing 1 mM PMSF (protease inhibitor). Equal amounts of denatured proteins from each sample were subjected to 12% sodium SDS-PAGE gels, and then transferred to polyvinylidene fluoride membranes (Millipore, Burlington, MA, USA). After blocking, the membranes were incubated with primary antibodies overnight at 4 °C: Bax (CST, Beverly, MA, USA), Bcl2 (Abcam, Cambridge, UK), Claudin1 (Abcam, Cambridge, UK), Claudin5 (CST, Beverly, MA, USA), Occludin (Abcam, Cambridge, UK), ZO-1 (Thermo Fisher Scientific, MA, USA), β-catenin (CST, Beverly, MA, USA), iNOS (Abcam, Cambridge, UK) and β-actin (Abcam, Cambridge, UK). The protein bands were visualized on an image system (Tanon, Shanghai, China) and the quantitative analysis of the bands was performed by ImageJ software version 1.53.

### 2.15. Statistical Analysis

Significances were analyzed by one-way analysis of variance (ANOVA) with Tukey’s multiple comparisons test or student’s *t*-test using SPSS v24 (SPSS Inc., Chicago, IL, USA); statistical graphs were visualized by GraphPad Prism v8.0 (GraphPad Software, San Diego, CA, USA).

## 3. Results

### 3.1. Aggregation Evaluation of Lactic Acid Bacteria

**Figure 1:**
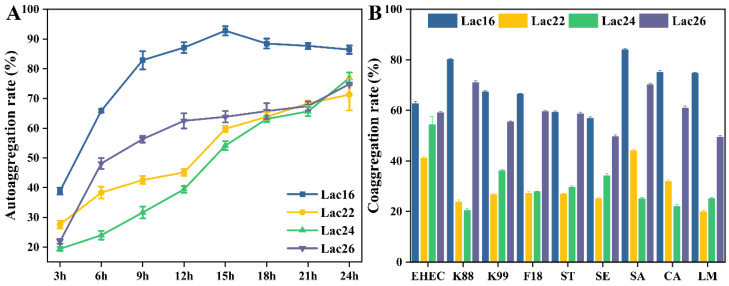
Auto-aggregation rate (**A**) and co-aggregation rate of lactic acid bacteria with zoonotic pathogens (**B**). Results are mean ± standard deviation for three independent experiments.

As shown in Figure 1A, the auto-aggregation rate of the lactic acid bacteria strains gradually increased with time, reaching over 70% at 24 h, and Lac16 exhibited a higher (*p* < 0.05) auto-aggregation rate than the others (Lac22, Lac24, Lac26). In addition, the co-aggregation rates with pathogens (EHEC, K88, K99, F18, ST, SE, SA, LM, CA) of Lac16 and Lac26 were higher (*p* < 0.05) than Lac22 and Lac24 (Figure 1B).

### 3.2. Tolerance of Lactic Acid Bacteria to Simulated Gastrointestinal Juices and Bile Salts

**Figure 2:**
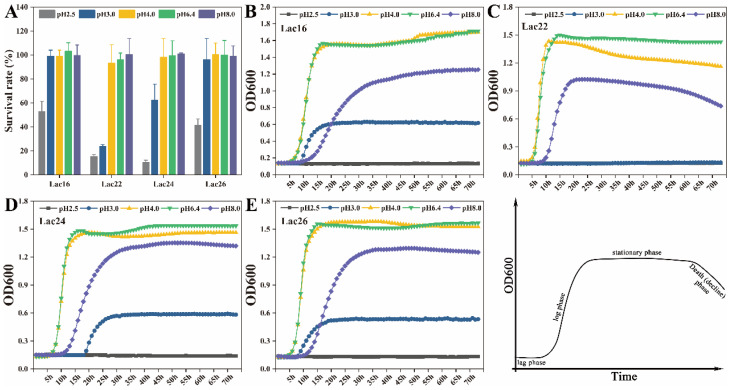
Survival rate (**A**) and growth curves (**B**–**E**, OD600 value) of lactic acid bacteria in simulated gastrointestinal juices at different pH values. Results are mean ± standard deviation for three independent experiments.

These four lactic acid bacteria strains (Lac16, Lac22, Lac24, Lac26) exhibited good tolerance to the simulated intestinal juices at pH 8.0. Lac16 and Lac26 exhibited better tolerance to the simulated gastric juices with almost 100% survival rate at pH 3.0 and pH 4.0, while with 40–55% at pH 2.5 (Figure 2A). The survival rate of Lac24 was 62.28% at pH 3.0 (Figure 2A). Additionally, these four lactic acid bacteria strains could grow well at pH 4.0 and pH 8.0, with 0.9–1.8 absorbance values at stationary phase (Figure 2B–E). Lac16, Lac24 and Lac26 could grow at pH 3.0 with 0.3–0.6 absorbance values at stationary phase (Figure 2B–E). The log phases and stationary phases of these four lactic acid bacteria strains were delayed at pH 3.0 and pH 8.0 compared with these at pH 4.0 and pH 6.4 (Figure 2B–E).

The lag time of these four strains in different concentration of porcine bile were ranged from −7.11 h to 2.55 h. Lac16 and Lac26 strains exhibited better bile tolerance with the lag time of 0.22 h~−7.11 h than Lac22 and Lac24 with the lag time of 0.28 h~−3.56 h (Table 1).

### 3.3. Antimicrobial Activity of Lactic Acid Bacteria

**Figure 3:**
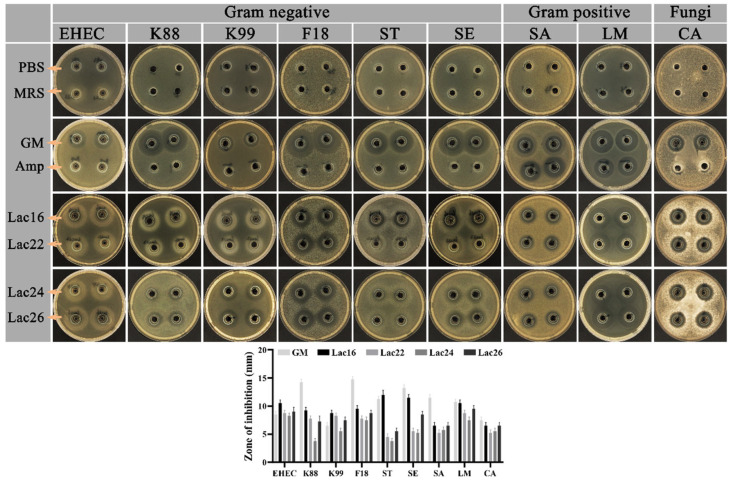
Inhibitory effect of fermented supernatant of lactic acid bacteria on the growth of zoonotic pathogens. Results are mean ± standard deviation for four independent experiments.

As shown in Figure 3, all selected pathogens were sensitive to 100 μg/mL gentamicin, while some gram-negative zoonotic pathogens (e.g., EHEC, K88, K99, F18, ST, SE) showed antimicrobial resistance to 100 μg/mL ampicillin. The fermented supernatants of four lactic acid bacteria could inhibit the growth of gram-negative (EHEC, K88, K99, F18, ST, SE), gram-positive (SA, LM), and fungal (CA) pathogens (Figure 3).

### 3.4. Lactic Acid Production of Lactic Acid Bacteria

**Figure 4:**
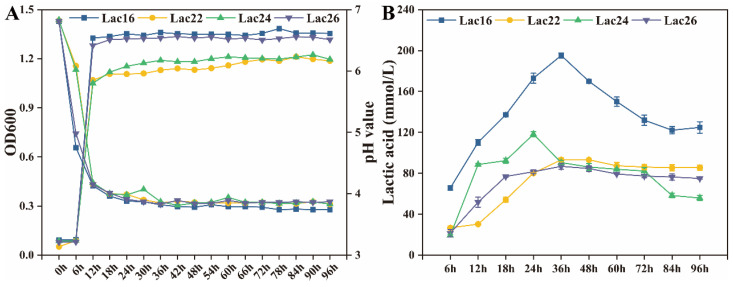
The growth (**A**), pH (**A**) and lactic acid production (**B**) of lactic acid bacteria. Results are mean ± standard deviation for three independent experiments.

As shown in Figure 4, the pH values of these four strains fermented cultures were ~3.8 at stationary phases. The lactic acid production of these four strains was increased at the log phases and early stationary phases, and decreased at the late stationary phases. Lac16 produced higher (*p* < 0.05) lactic acid than the other three strains. These four lactic acid bacteria were all identified as *Lactiplantibacillus plantarum* based on 16S rDNA gene sequences (Figure S1).

### 3.5. Lac16 Inhibited Growth and Biofilm Formation of Zoonotic Pathogens

**Figure 5:**
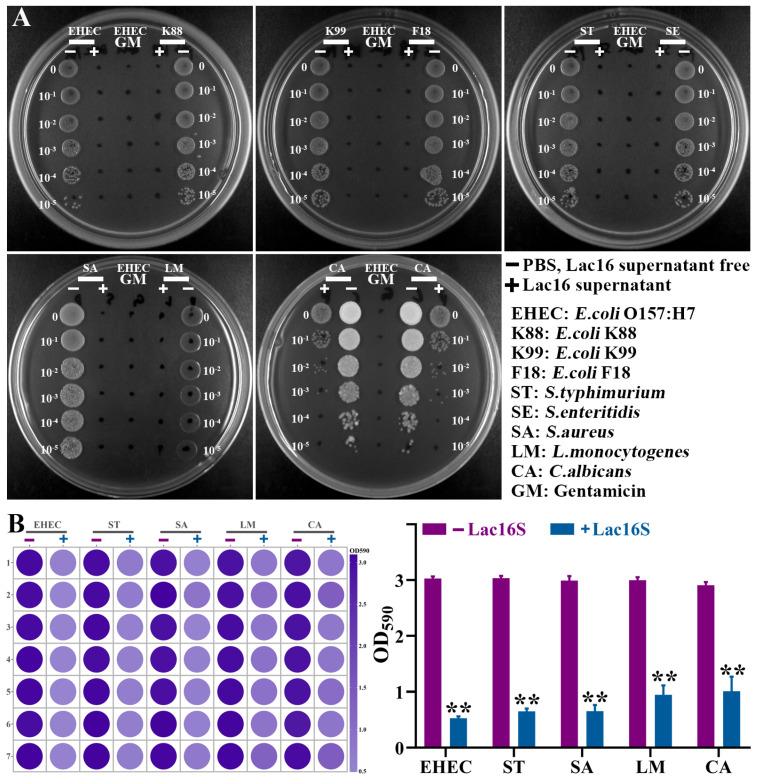
Effect of *L. plantarum* Lac16 on the growth (**A**) and biofilm formation (**B**) of zoonotic pathogens. Results are mean ± standard deviation for seven independent experiments. Significant differences are indicated by ** *p*< 0.01.

All results mentioned above indicated that Lac16 strain exhibited the best potential probiotic traits. The antimicrobial activity assay was performed to further confirm that the fermented supernatants of Lac16 could significantly inhibit the growth of gram-negative (EHEC, K88, K99, F18, ST, SE), gram-positive (SA, LM) and fungal (CA) zoonotic pathogens (Figure 5A). Moreover, Lac16 fermented supernatant could significantly (*p* < 0.05) inhibit the biofilm formation of gram-negative (EHEC, ST), gram-positive (SA, LM) and fungal (CA) pathogens (Figure 5B).

### 3.6. Lac16 Inhibited the Gene Expression of Key Virulence Traits of EHEC

**Figure 6:**
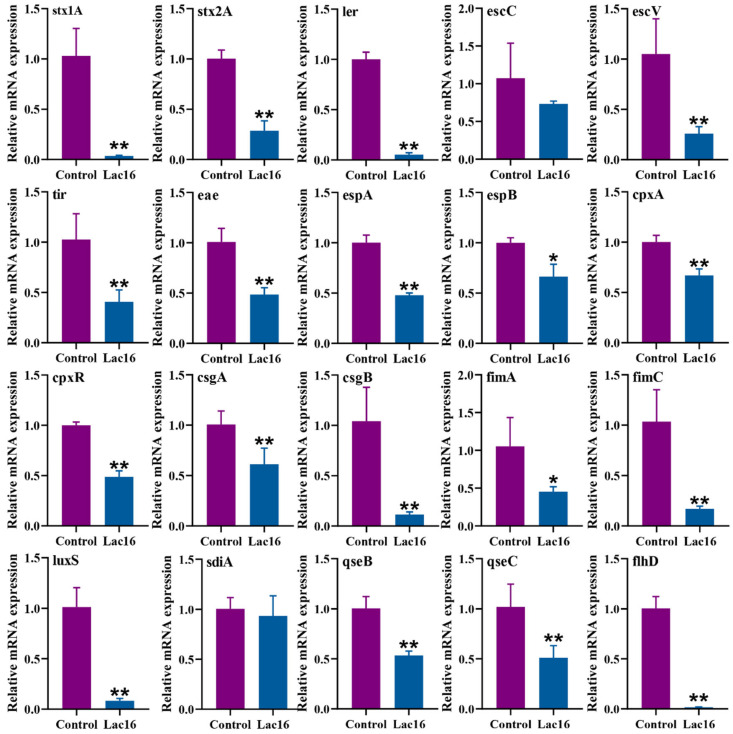
Effect of the fermented supernatants of *L. plantarum* Lac16 on gene expression of key virulence traits of EHEC. Results are mean ± standard deviation for three independent experiments. Significant differences are indicated by * *p* < 0.05 and ** *p* < 0.01.

As shown in Figure 6, the fermented supernatants of Lac16 significantly (*p* < 0.05 or *p* < 0.01) inhibited the mRNA expressions of the locus of enterocyte effacement (LEE) encoded virulence genes (ler, escV, tir, eae, espA, espB), lethal phage-encoded virulence factor Shiga toxin (stx1A, stx2A), flagella biogenesis and motility related genes (qseC, qseB, flhD) in EHEC. Moreover, the mRNA expressions of the antibiotic resistance related genes (cpxA, cpxR), biofilm formation related curli genes (csgA, csgB), AI-2 quorum sensing gene (luxS) and type I fimbriae genes (fimA, fimC) were also significantly (*p* < 0.05 or *p* < 0.01) reduced in EHEC treated with the fermented supernatants of Lac16.

### 3.7. Lac16 Protected C. elegans against Pathogen Infection

**Figure 7:**
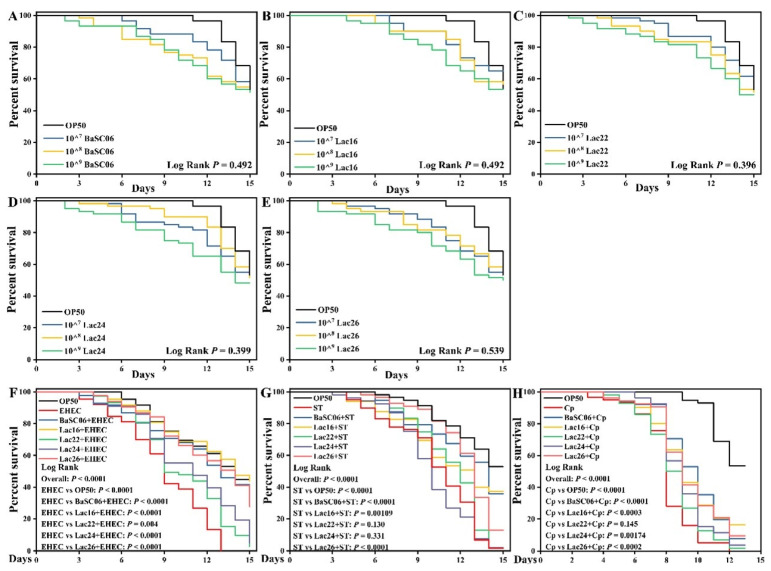
Effect of probiotics on the life-span of *C. elegans* uninfected or infected with zoonotic pathogens. (**A**–**E**) L4-stage worms were transferred to lawns of bacteria (lactic acid bacteria, BaSC06, *E. coli* OP50) grown on NGM plates (30 worms/plate) with 3 plates per group for 15 days. (**F**–**H**) L4-stage worms were transferred to lawns of bacteria (lactic acid bacteria, BaSC06, *E. coli* OP50) grown on NGM plates (30 worms/plate) with 3 plates per group for colonization for 1 day. Then, the treated worms were washed and transferred to lawns of zoonotic pathogens (EHEC, ST, Cp) grown on NGM plates for infection for another day. Finally, the infected worms were washed and transferred to lawns of *E. coli* OP50 grown on NGM plates (Day 0).

*C. elegans* mimics many key aspects of animal intestinal physiology, indicating that *C. elegans* is an invaluable model organism to study host-microbe interactions. *C. elegans* was selected as a live model host to further evaluate the protective effect of Lac16. As shown in Figure 7A–E, the life span of *C. elegans* reared on probiotic lawns (BaSC06, Lac16, Lac22, Lac24, Lac26) in a dose-dependent manner was similar to worms fed *E. coli* OP50 (*p* > 0.05), indicating that the probiotics do not affect the normal development of the worms. Furthermore, *C. elegans* survivals were significantly (*p* < 0.01) increased in worms fed probiotic lawns (BaSC06, Lac16, Lac26) before infection with pathogens (EHEC, ST, Cp) as compared with worms fed *E. coli* OP50 (Figure 7F–H). In addition, Lac24 significantly (*p* < 0.01) protected *C. elegans* against EHEC and Cp infection (Figure 7F, H), and Lac22 significantly (*p* < 0.01) protected *C. elegans* against EHEC infection (Figure 7F).

### 3.8. Lac16 Promoted the Wound Closure of Intestinal Epithelial Cells

**Figure 8:**
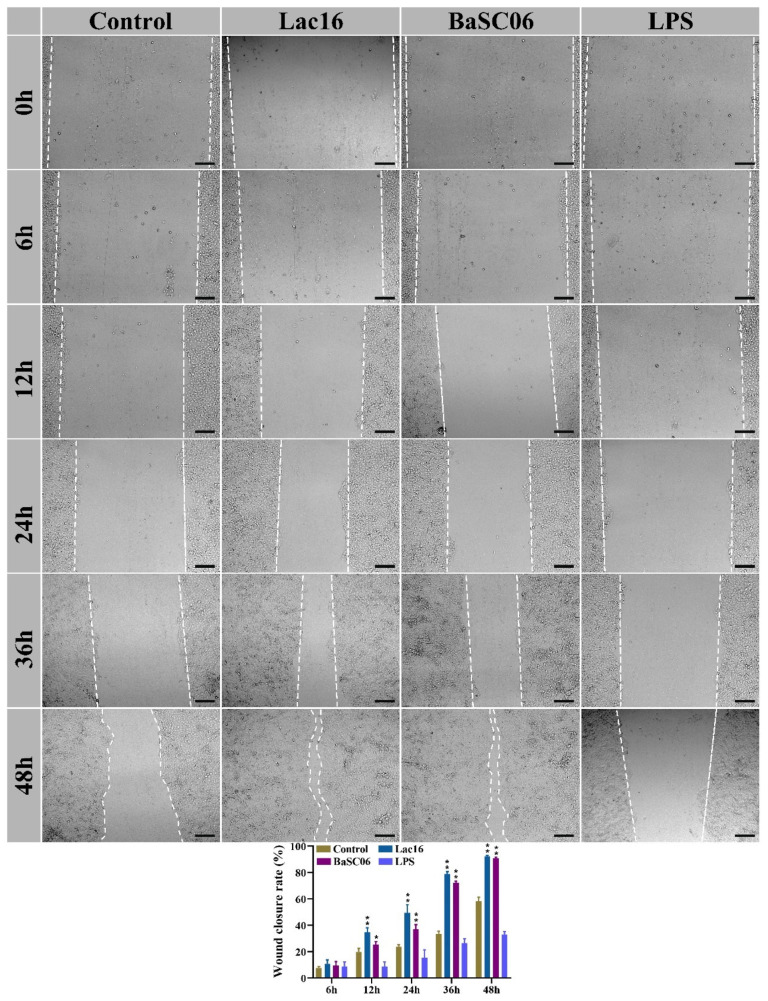
Probiotics promote the wound closure of IPEC-J2 cells. IPEC-J2 cells were treated with probiotics (MOI = 100) or LPS (40 µg/mL) for different times. Results are mean ± standard deviation for three independent experiments. Significant differences versus Control group: * *p* < 0.05; ** *p* < 0.01.

Since the intestinal epithelial barrier dysfunction caused by the infection of zoonotic pathogens contributes to *C. elegans* death, IPEC-J2 epithelial cell derived from porcine jejunum was selected as an in vitro model to further elucidate the underlying protective mechanism of Lac16 acting as probiotics. As shown in Figure 8, the wound closure of IPEC-J2 cells incubated with Lac16 and BaSC06 was significantly (*p* < 0.05 or *p*< 0.01) promoted, with the wound closure rate from 34.62% (Lac16) or 25.49% (BaSC06) at 12 h to 92.27% (Lac16) or 90.61% (BaSC06) at 48 h. Additionally, LPS treatment significantly (*p* < 0.05 or *p* < 0.01) delayed cell wound closure from 12 h to 48 h.

### 3.9. Lac16 Ameliorated LPS-Induced Apoptosis of Intestinal Epithelial Cells

**Figure 9:**
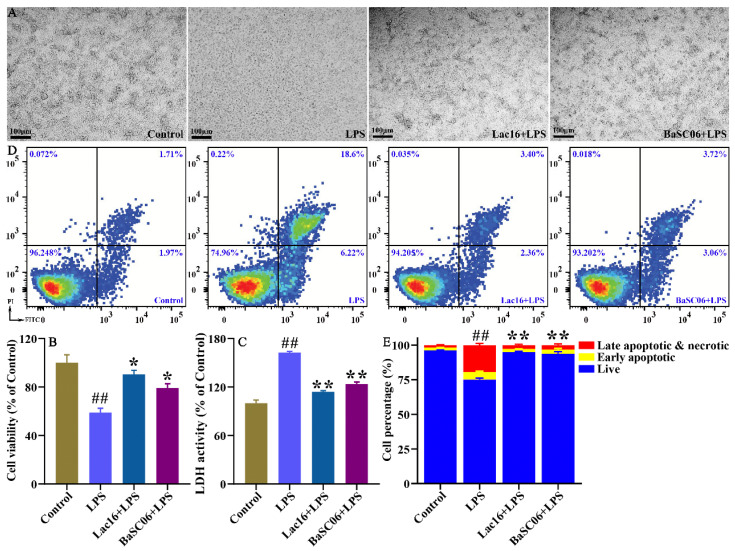
Probiotics attenuated LPS-induced cytotoxicity in IPEC-J2 cells. IPEC-J2 cells were pre-incubated with probiotics (MOI = 100) for 12 h and then treated with LPS (40 µg/mL) for 12 h. (**A**) The representative IPEC-J2 cell images. (**B**) The cell viability of IPEC-J2 cells. (**C)** The LDH activity in the supernatant of the treated cells. (**D**,**E**) Flow cytometry analysis of Annexin V-FITC/PI staining apoptotic cells. Results are mean ± standard deviation for three independent experiments. Significant differences versus Control group: ## *p* < 0.01. Significant differences versus LPS group: * *p* < 0.05; ** *p* < 0.01.

LPS, derived from gram-negative bacteria, significantly induces intestinal epithelial barrier dysfunction by eliciting inflammatory responses, inducing cell apoptosis, and downregulating expression of tight junction proteins [31,32], and is often used as a model to study pathogen-host interplay. In the present study, compared with the Control group, LPS treatment obviously induced IPEC-J2 cell damage as evidenced by the unattached cells, the fragmented and pyknotic nuclei (Figure 9A). Moreover, LPS significantly (*p* < 0.01) decreased cell viability (Figure 9B) and increased LDH release from the damaged IPEC-J2 cells (Figure 9C). The results of flow cytometry analysis further showed that LPS treatment significantly (*p* < 0.01) decreased the proportion of live IPEC-J2 cells and significantly (*p* < 0.01) increased proportions of early and late apoptotic cells and necrotic cells (Figure 9D,E). However, Lac16 or BaSC06 could significantly (*p* < 0.05 or *p* < 0.01) alleviate LPS-induced cytotoxicity and cell apoptosis (Figure 9).

**Figure 10:**
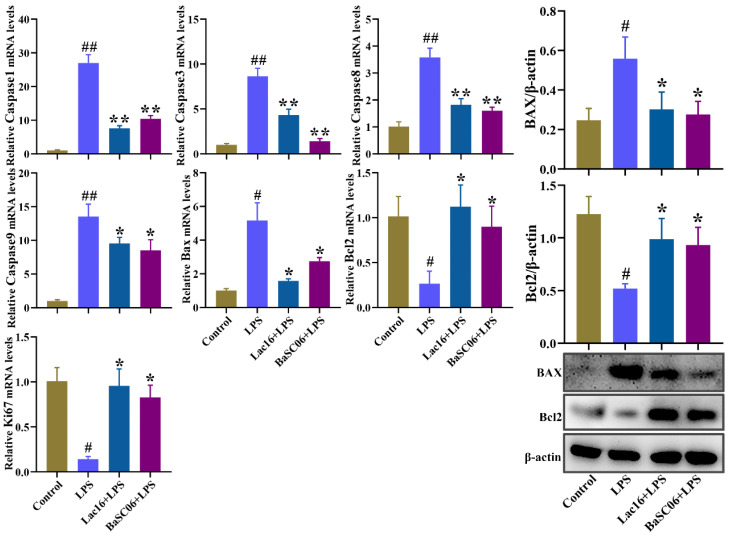
Effect of probiotics on the expression of cell apoptosis related genes and proteins in IPEC-J2 cells. IPEC-J2 cells were pre-incubated with probiotics (MOI = 100) for 12 h and then treated with LPS (40 µg/mL) for 12 h. Results are mean ± standard deviation for three independent experiments. Significant differences versus Control group: # *p* < 0.05; ## *p* < 0.01. Significant differences versus LPS group: * *p* < 0.05; ** *p* < 0.01.

Additionally, LPS treatment significantly induced IPEC-J2 cell apoptosis as evidenced by the significantly (*p* < 0.05 or *p* < 0.01) upregulated mRNA expression of cell apoptosis related genes (caspase1, caspase3, caspase8, caspase9, BAX) and BAX protein, and the significantly (*p* < 0.01) downregulated mRNA expression of cell proliferation related genes (Ki67, Bcl2) and Bcl2 protein, which could be significantly (*p* < 0.05 or *p* < 0.01) ameliorated by Lac16 or BaSC06 pre-incubations (Figure 10).

### 3.10. Lac16 Ameliorated LPS-Induced Intestinal Epithelial Barrier Dysfunction

**Figure 11:**
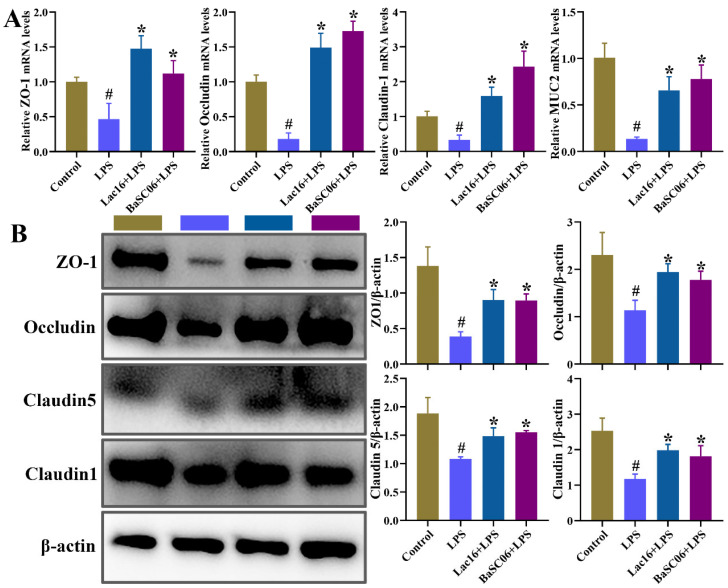
Effect of probiotics on the expression of tight junction proteins in IPEC-J2 cells. IPEC-J2 cells were pre-incubated with probiotics (MOI = 100) for 12 h and then treated with LPS (40 µg/mL) for 12 h. The mRNA (**A**) and protein (**B**) expression of tight junction proteins. Results are mean ± standard deviation for three independent experiments. Significant differences versus Control group: # *p* < 0.05. Significant differences versus LPS group: * *p* < 0.05.

The epithelial barrier function of IPEC-J2 was disrupted by LPS treatment, as illustrated by the significantly (*p* < 0.05) decreased expression of tight junction-related mRNA genes (ZO-1, Occludin, Claudin1, MUC2) and proteins (ZO-1, Occludin, Claudin5, Claudin1), which was significantly (*p* < 0.05 or *p* < 0.01) ameliorated by Lac16 or BaSC06 pre-incubations (Figure 11).

### 3.11. Lac16 Activated LPS-Induced Inhibition of Wnt/β-Catenin Signaling Pathway of Intestinal Epithelial Cells

**Figure 12:**
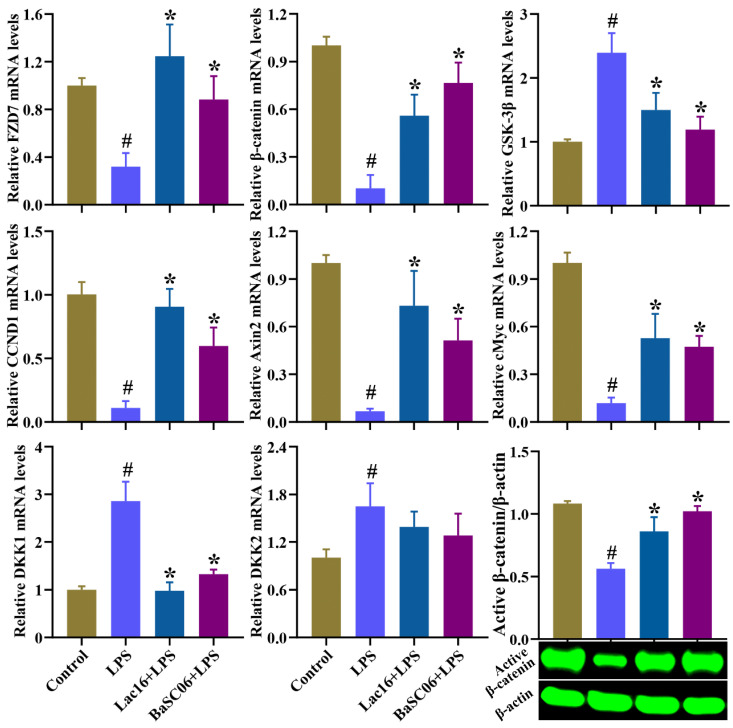
Effect of probiotics on the expression of Wnt/β-catenin signaling pathway in IPEC-J2 cells. IPEC-J2 cells were pre-incubated with probiotics (MOI = 100) for 12 h and then treated with LPS (40 µg/mL) for 12 h. Results are mean ± standard deviation for three independent experiments. Significant differences versus Control group: # *p* < 0.05. Significant differences versus LPS group: * *p* < 0.05.

As shown in Figure 12, compared with the Control group, LPS treatment significantly (*p* < 0.05) inhibited the expression of FZD7, β-catenin and target genes (CCND1, Axin2, cMyc), and significantly (*p* < 0.05) induced expression of GSK-3β, DKK1 and DKK2 in IPEC-J2 cells, whereas these side effects could be inverted by Lac16 or BaSC06 pre-treatments, except for DKK2 gene expression.

### 3.12. Lac16 Inhibited LPS-Induced Inflammatory Responses of Intestinal Epithelial Cells

**Figure 13:**
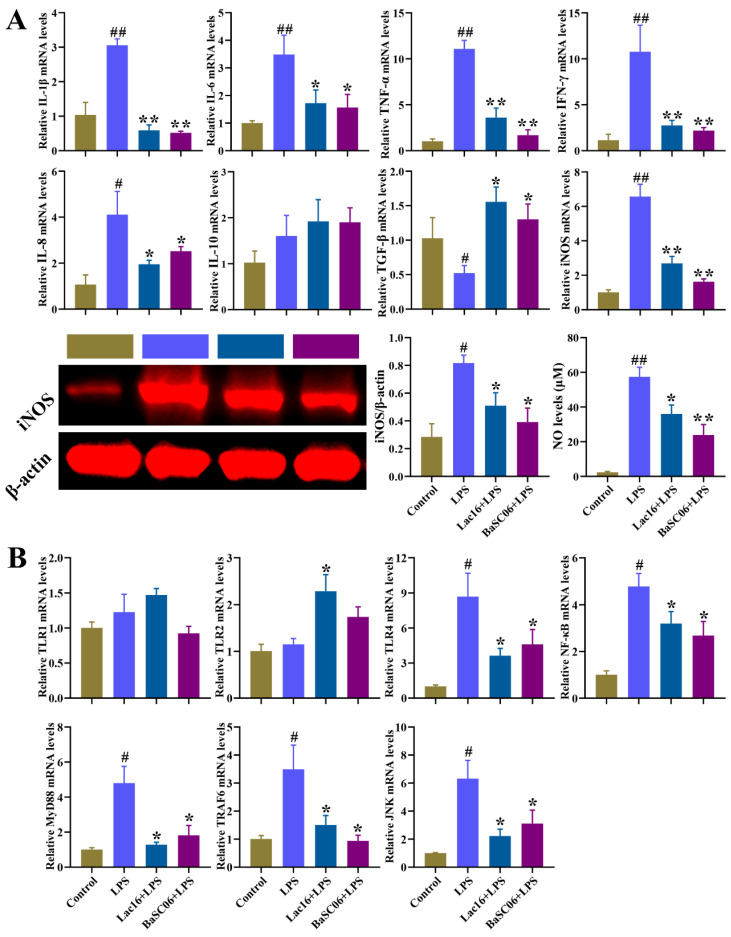
Effect of probiotics on the inflammatory responses of LPS-treated IPEC-J2 cells. The expression of inflammatory factors (**A**) and TLRs signaling pathway (**B**) in IPEC-J2 cells. IPEC-J2 cells were pre-incubated with probiotics (MOI = 100) for 12 h and then treated with LPS (40 µg/mL) for 12 h. Results are mean ± standard deviation for three independent experiments. Significant differences versus Control group: # *p* < 0.05; ## *p* < 0.01. Significant differences versus LPS group: * *p* < 0.05; ** *p* < 0.01.

Compared with the Control group, LPS significantly (*p* < 0.05 or *p* < 0.01) increased the mRNA expression of pro-inflammatory cytokines (IL-1β, IL-6, TNF-α, IFN-γ, IL-8) and significantly (*p* < 0.05) decreased TGF-β mRNA expression (Figure 13A). Moreover, iNOS expression and NO release were also significantly (*p* < 0.05 or *p* < 0.01) increased in LPS-treated IPEC-J2 cells. Compared with the LPS group, Lac16 or BaSC06 significantly (*p* < 0.05 or *p* < 0.01) inhibited LPS-induced upregulation of pro-inflammatory cytokines (IL-1β, IL-6, TNF-α, IFN-γ, IL-8), iNOS and NO release, and downregulation of TGF-β (Figure 13A). In addition, these results further showed that, compared with the Control group, LPS significantly (*p* < 0.05) increased the mRNA expression of TLR4, NF-κBp65, MyD88, TRAF6 and JNK genes, which could be inverted by Lac16 or BaSC06 pre-treatments (Figure 13B).

## 4. Discussion

Although many studies have shown that probiotics have antibacterial and antifungal activities [25,33], the underlying mechanism of probiotics in alleviating zoonotic pathogen infection-induced toxicity and damage to host tissues and cells remains unclear. Our previous studies reported that *B. amyloliquefaciens* BaSC06 could enhance the phagocytosis and bactericidal capacity of macrophages by inducing autophagy [34] and M1 macrophage polarization [35] to protect against pathogen infection. In the present study, four candidate probiotics were selected to assess their capability to alleviate zoonotic pathogen-induced damage and the underlying mechanisms. The auto-aggregation capacity of lactic acid bacteria reflects an adhesion capability to gastrointestinal epithelium [26], while the co-aggregation of lactic acid bacteria with pathogens reflects the capacity to form a barrier that prevents pathogen colonization [36]. The high auto-aggregation and co-aggregation activities of these four lactic acid bacteria strains may reflect their abilities to adhere to intestinal epithelium against colonization of pathogens [26]. Although Lac16 and Lac26 exhibited high auto-aggregation and co-aggregation abilities, whether the high auto-aggregation abilities of these two strains contribute the enhanced co-aggregation or not remains unclear and needs further investigation. It is reported that auto-aggregation is generally mediated by autoagglutinins (e.g., proteins and exopolysaccharides) of *Lactobacillus* strains [37]. Coaggregation among different bacterial species is typically mediated by protein adhesins on one cell type and complementary saccharide receptors on the other [38]. We speculate that Lac16 and Lac26 with high auto-aggregation and co-aggregation activities might be due to the two strains expressing more autoagglutinins and adhesins, although more direct evidences are needed. The simulated gastrointestinal juices and bile tolerances are crucial properties for probiotic strains to survive and grow in the gastrointestinal tract [26,27]. The present study showed that Lac16 and Lac26 strains could tolerate low pH 2.5 and bile, suggesting their stress resistance in the gastrointestinal tract and their potential as probiotic strains, which was consistent with previous studies [26,39,40].

As a potential advanced alternative to antimicrobials, many studies have shown that lactic acid bacteria strains exert beneficial effects on inhibiting growth and toxin secretion of pathogens by secreting organic acids and bacteriocins [25,41]. This study demonstrated that these four candidate probiotics could inhibit pathogens’ growth, among which Lac16 produced the highest level of lactic acid. Biofilm of pathogens plays important roles in tolerance or resistance to antimicrobials, antimicrobial agents or host defense [42,43], and pathogens can induce toxicity and damage to the host by releasing toxins or virulence factors [19], which are regulated by quorum-sensing signaling [44,45]. The current study further showed that Lac16 not only inhibited multiple drug resistant pathogens, but also significantly inhibited biofilm formation of enterohemorrhagic *E. coli* O157:H7, and repressed the mRNA expression of key virulence traits, indicating that Lac16 has a great potential to prevent zoonotic pathogen infection and to reduce the contamination of zoonotic pathogens in raw food products. *C. elegans* mimics many key aspects of animal and human intestinal physiology, indicating that *C. elegans* can be an invaluable model organism to study host-microbe interactions [46]. In this study, enterohemorrhagic *E. coli* O157:H7 and *S. typhimurium* were selected as representative strains of gram-negative enteric pathogens (*Escherichia* and *Salmonella*); *C. perfringens* was selected as a representative strain of gram-positive enteric pathogens to infect *C. elegans*. The current study showed that these four candidate probiotics were benign and did not affect the normal development of *C. elegans*, and that probiotics (BaSC06, Lac16, Lac26) pre-treatment protected *C. elegans* against zoonotic pathogen (EHEC, ST, Cp) infection-induced deaths, indicating that Lac16 exerts great protective phenotypes against infection of enteric pathogens.

Probiotics exert protective phenotypes not only by directly exhibiting antibacterial and antifungal activities, but also by interacting with host tissues or cells to modulate gastrointestinal epithelial barrier functions and the immune system [25]. As the first physical defensive barrier of the gastrointestinal tract, intestinal epithelial cells play crucial roles in inhibiting the invasion of enteric pathogens and maintaining gut homeostasis [16]. To mimic enteric pathogen infection-induced epithelial damage in vivo, the in vitro wound scratch assay in a IPEC-J2 epithelial cell was employed, and the present results demonstrated that Lac16 and BaSC06 treatments significantly promoted cell epithelial repair, whereas LPS exposure delayed cell epithelial repair, indicating that Lac16 exerts beneficial effects in promoting intestinal epithelial repair, consistent with a previous study [47]. To mimic enteric pathogens-induced epithelial dysfunction in vivo, LPS exposure-induced intestinal epithelial barrier damage and immune responses were selected as an in vitro model to further elucidate the underlying protective mechanism of Lac16 acting as probiotics. Our results showed that Lac16 and BaSC06 pre-treatments significantly ameliorated LPS-induced intestinal epithelial damage, as illustrated by the reduced cell cytotoxicity, epithelial death and apoptosis of IPEC-J2 cells. It is reported that intestinal epithelial cell apoptosis and death often result from enteric infections and are key contributing factors towards intestinal epithelial barrier dysfunction [19]. The present study demonstrated that Lac16 and BaSC06 strains significantly alleviated LPS exposure-induced intestinal epithelial barrier dysfunction as evidenced by the upregulation of tight junction proteins, which might be related to the beneficial changes in cell death and apoptosis mentioned above. Evidence has shown that the Wnt/β-catenin signaling pathway is central to the developmental and disease-related cellular processes of hosts [48,49], and plays an important role in epithelial cell proliferation, regeneration and repair [49,50,51]. Further, Wnt/β-catenin signaling is also reported to reduce Bax-mediated apoptosis and thereby promote cell survival [52,53]. The present results demonstrated that LPS inhibited the activation of the Wnt/β-catenin pathway, consistent with a previous study [54]. However, the inhibited Wnt/β-catenin signaling pathway induced by LPS could be markedly activated by Lac16 and BaSC06 pre-treatments. These above results indicate that Lac16 might ameliorate LPS induced cytotoxicity, cell apoptosis and barrier dysfunction through the Wnt/β-catenin signaling pathway.

Immune responses are regulated by complex and cross-linked endogenous cellular signaling pathways and their modulators [55], and are initiated immediately after pattern-recognition receptors (PRRs) recognize pathogen-associated molecular patterns (PAMPs), which has a critical role in both innate and adaptive immune responses against enteric pathogens and inhibiting enteric infections [56]. Evidence has shown that host-microbe interactions depend on the PRRs (e.g., Toll-like receptors (TLRs), Nod-like receptors (NLRs) and RIG-I-like receptors (RLRs)), and TLR4 specifically recognizes bacteria-derived lipopolysaccharide (LPS) [55,57]. After recognition, TLRs trigger the activation of downstream signaling pathways (e.g., MAPK and NF-κB) via MyD88-dependent and TRIF-dependent pathways, which results in the secretion of inflammatory cytokines and chemokines [55,57]. The current study demonstrated that Lac16 and BaSC06 pre-treatments markedly inhibited LPS-induced activation of TLR4-MyD88-dependent signaling pathway, as evidenced by the upregulation of TLR4, NF-κBp65, MyD88, TRAF6 and JNK genes. The activation of TLR4-MyD88-dependent signaling pathway induced the production of inflammatory cytokines (e.g., IL-1, IL-6, IL-12, TNF-α, IFNs), which are necessary for enteric pathogen clearance [58]. However, excessive inflammatory responses provoked by enteric pathogens can detrimentally damage host tissues or cells [19,59]. Therefore, the exaggerated inflammation is tightly controlled by associated negative feedback loops and anti-inflammatory cytokines (e.g., IL-10, TGF-β) [60,61]. Our results demonstrated that Lac16 and BaSC06 strains significantly attenuated LPS-induced inflammatory responses by inverting the upregulation of pro-inflammatory properties (IL-1β, IL-6, TNF-α, IFN-γ, IL-8, iNOS, NO release) and the downregulation of TGF-β, which may contribute to the reduced cell death and apoptosis in probiotics-treated intestinal epithelial cells mentioned above. These results indicate that Lac16 alleviates LPS-induced inflammatory responses mediated by TLR4/MyD88 signaling pathway.

## 5. Conclusions

**Figure 14:**
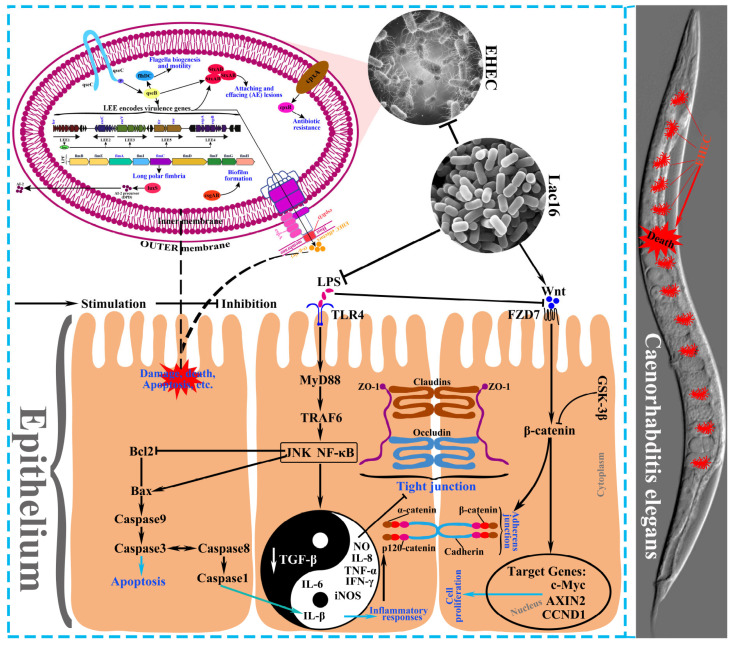
Graphical summary of the protective effect of *Lactiplantibacillus plantarum* Lac16 against enterohemorrhagic *E. coli* O157:H7 infection. *L. plantarum* Lac16 attenuated enterohemorrhagic *E. coli* O157:H7 infection-induced toxicity to *C. elegans* and LPS exposure-induced intestinal epithelial damage by inhibiting key virulence traits of *E. coli* O157:H7 and enhancing intestinal epithelial barrier function, which might be mediated by the activated Wnt/β-catenin signaling pathway and the inhibited TLR4-MyD88 signaling pathway.

The current results demonstrate that *L. plantarum* 16 could directly inhibit the biofilm formation and growth of enterohemorrhagic *E. coli* O157:H7 by inhibiting the mRNA expression of key virulence traits via quorum-sensing signaling. Meanwhile, *L. plantarum* 16 significantly ameliorated LPS-induced intestinal epithelial barrier dysfunction by promoting epithelial repair, attenuating epithelial cell apoptosis and death, and attenuating inflammatory responses, which may be mediated by the activated Wnt/β-catenin signaling pathway and the inhibited TLR4-MyD88 signaling pathway. All told, *L. plantarum* 16 protected *C. elegans* against enterohemorrhagic *E. coli* O157:H7 infection induced death by the ways mentioned above (Figure 14). However, further investigations in animal models (e.g., mice, pig, broilers) should be conduct to convincingly support the beneficial effects of *L. plantarum* Lac16 on protecting against enterohemorrhagic *E. coli* O157:H7 infection.

## Figures and Tables

**Table 1:** Bile tolerance of lactic acid bacteria based the time delay method.

	^Strains^	Lac16	Lac22	Lac24	Lac26
_Oxgall (%)_					
Time to reach OD_620_ of 0.3 units (h)					
0%		9.06 ± 0.08	6.50 ± 0.14	7.83 ± 0.13	8.94 ± 0.08
0.10%		6.56 ± 0.08	6.78 ± 0.21	6.67 ± 0.13	9.17 ± 0.13
0.20%		5.00 ± 0.14	6.56 ± 0.08	6.39 ± 0.21	9.56 ± 0.08
0.30%		3.22 ± 0.08	4.22 ± 0.08	5.28 ± 0.16	3.89 ± 0.21
0.40%		1.94 ± 0.08	3.22 ± 0.08	4.28 ± 0.21	2.50 ± 0.14
Lag time (h)					
0.10%		−2.50 ± 0.08 ^c^	0.28 ± 0.21 ^a^	−1.17 ± 0.13 ^b^	0.22 ± 0.13 ^a^
0.20%		−4.06 ± 0.14 ^d^	0.06 ± 0.08 ^b^	−1.44 ± 0.21 ^c^	0.61 ± 0.08 ^a^
0.30%		−5.83 ± 0.08 ^c^	−2.28 ± 0.08 ^a^	−2.55 ± 0.16 ^a^	−5.05 ± 0.21 ^b^
0.40%		−7.11 ± 0.08 ^c^	−3.28 ± 0.08 ^a^	−3.56 ± 0.21 ^a^	−6.44 ± 0.14 ^b^

Results are mean ± standard deviation for three independent experiments. Different lowercase letters indicate a significant difference (*p* < 0.05).

## Data Availability

The raw data presented in this study are available on request from the corresponding author.

## Supplementary Materials

The following supporting information can be downloaded at: https://www.mdpi.com/article/10.3390/cells12101438/s1, Table S1: List of real-time PCR primers for EHEC bacteria; Table S2: List of real-time PCR primers for IPEC-J2; Figure S1: Phyloge-netic tree of probiotics. The tree was constructed using software MEGA 10.1.8 by neighbor-joining method based on 16S rDNA gene sequences with 1000 replications in bootstrap test.
