# Supplementary material for: Lactiplantibacillus plantarum Lac16 Attenuates Enterohemorrhagic Escherichia coli O157:H7 Infection by Inhibiting Virulence Traits and Improving Intestinal Epithelial Barrier Function
Source: Cells. 2023 May 21;12(10):1438. doi: 10.3390/cells12101438 (PMC10217168; doi:10.3390/cells12101438)
Supplement: Supplementary file 1 [file cells-12-01438-s001.zip › cells-2299035-supplementary.pdf]

Supplementary Material

Table S1. List of real-time PCR primers for EHEC bacteria

Gene name	Primer sequence (5′-3′)	Product size
rpoA	F:GTGACCCTTGAGCCTTTAGAG<br>R:ACACCATCAATCTCAACCTCG	116
16S rRNA	F:GAAAGCGTGGGGAGCAAAC<br>R:ACATGCTCCACCGCTTGTG	188
stx1A	F:CCCACGGACTCTTCCATCTG<br>R:ATTCTGGGAAGCGTGGCATT	114
stx2A	F:TGTCTGAAACTGCTCCTGTG<br>R:GATATTCTCCCCACTCTGACAC	127
cpxA	F:TGGTGAAAGCAAGGAACTGG<br>R:TGATTGGCTTTGATGGTCTCG	132
cpxR	F:GATGACTATCTCCCGAAACCG<br>R:ATCAACTTCCAGTGTCGGTG	129
tir	F:GAGGGAGTCAAATAGCGGTG<br>R:ATCTGAACGAAGGCTGGAAG	76
ler	F:CGAGTCCATCATCAGGCACA<br>R:TACAGCAGGAAGCAAAGCGA	132
eae	F:CATCACCACCGCTTGCTTTC<br>R:GGGCTGAGGTTAAAGCGACT	150
espA	F:AGCTATTTGAGGAACTCGGTG<br>R:CATCTTTTGTGCCGTGGTTG	140
espB	F:GGTCAAGGCTACGGAAAGTG<br>R:TCTTCAGCAAAGTCAGAGGC	144
escC	F:CTGAAGACAATGGCAAGTAATGG<br>R:ACTGCATTAAGACGTGGATCAG	119
escV	F:GAGTGCAAAAGGAAAGCCAG<br>R:ATGATACCAGCAATAGCGTCC	82
csgA	F:GGCAGGTGTTGTTCCTCAGT<br>R:AAAGCCACGTTGGGTCAGAT	238
csgB	F:GGTAGTAGCAACCGGGCAAA<br>R:GCACCTTGCGAAATACTGGC	101
fimA	F:CCAGTTCTGCTGTCGGTTTT<br>R:TCAGGGTTGTTTGCTCACTG	231
fimC	F:GACACTTCACCTTTTGGCGG<br>R:CAATGGCACCTGAAGAAGCG	160
luxS	F:CATACCCTGGAGCACCTGTT<br>R:TGATCCTGCACTTTCAGCAC	191
sdiA	F:AGACTTTCGCGCACCAGTAA	181

	R:AAGCACAGCCGTTATGGGAA	
qseB	F:TGAGCCGGTACTGATCCTGA<br>R:GCCCTTCTACACGTTCCTCC	56
qseC	F:CCGGACAACCGCTATTGCTA<br>R:CCTTCGCCTCAAATCCACCT	300
flhD	F:CAGTGCCGCTAATGTTGTCG<br>R:TCCGCTATGTTTCGTCTCGG	57

Table S2. List of real-time PCR primers for IPEC-J2

Gene Name	Primers （5’-3’）	Accession no.
Caspase-1	F:AATCTCACCGCTTCGGACAT<br>R:TCCATACGACCCCTTGCTTC	NM_214162
Caspase-3	F:CGAGGCACAGAATTGGACTG<br>R:CCAGGAATAGTAACCAGGTGCTG	NM_214131
Caspase-8	F:ACCAGGTCGGAATTGAAGGAC<br>R:GCAAGGTCATGTCATCATCCAG	NM_001031779
Caspase-9	F:CAACCTGACTGCCAAGCAAA<br>R:TGACAGCCGTGAGAGAGCAC	XM_003127618
Bax	F:CTACCAAGAAGTTGAGCGAGTGTCTC<br>R:GTGTCCACGGCTGCGATCATC	XM_003127290
Bcl-2	F:AGCAGGTATTGAACGAACTCTTCCG<br>R:AGGCTCTAGGTGGTCATTCAGGTAAG	NM_214285
Ki67	F:TTGTCCCTGAATCCGCAAGA<br>R:TTCTCTGGTTGCTTGGTTGC	NM_001101827
ZO-1	F:CTCTTGGCTTGCTATTCG<br>R:AGTCTTCCCTGCTCTTGC	XM_021098896
Claudin-1	F:TGCCCCCGAAAAACAACATC<br>R:CACATGAAAATGGCTTCCCTC	NM_001244539
Occludin	F:GGTAACTTGGAGGCGTCTTC<br>R:GTAGTCTGTCTCGTAATGGTCTTG	NM_001163647
MUC2	F:CGGTCAAGGACGACACCATC<br>R:TGTTCCACACGAGAGCAAGG	XM_021082584
IL-1β	F:AGAGGGACATGGAGAAGCGA<br>R:GCCCTCTGGGTATGGCTTT	NM_001302388
IL-6	F:ATCAGGAGACCTGCTTGATG<br>R:TGGTGGCTTTGTCTGGATTC	NM_001252429
TNF-α	F:CTGTAGGTTGCTCCCACCTG<br>R:CCAGTAGGGCGGTTACAGAC	NM_214022
IFN-γ	F:GAGCCAAATTGTCTCCTTCTAC<br>R:CGAAGTCATTCAGTTTCCCAG	NM_213948

IL-8	F:TCCTGCTTTCTGCAGCTCTC<br>R:GGGTGGAAAGGTGTGGAATG	NM_213867
IL-10	F:CTGCATCCACTTCCCAACCA<br>R:CCCATCACTCTCTGCCTTCG	NM_214041
TGF-β	F:GAAGCGCATCGAGGCCATTC<br>R:GGCTCCGGTTCGACACTTTC	NM_214015
iNOS	F:CATCACCACGCCTCCAACTCAG<br>R:AGTCTCAAGCCTCTGCCTCTCG	NM_001143690
TLR1	F:GTCAGTCAGCACCGCAGTAA<br>R:CAGACAAACTGGAGGGTGGT	NM_001031775
TLR2	F:TCACTTGTCTAACTTATCATCCTCT<br>R:TCAGCGAAGGTGTCATTATTGC	NM_213761
TLR4	F:GCCATCGCTGCTAACATCATC<br>R:CTCATACTCAAAGATACACCATCG	NM_001293316
MyD88	F:GCTGGAACAGACCAACTAT<br>R:TCCTTGCTTTGCAGGTAAT	NM_001099923
TRAF6	F:GGGAACGATACGCCTTACAA<br>R:CTCTGTCTTAGGGCGTCCAG	NM_001105286
JNK	F:TGCTTTGTGGAATCAAGCAC<br>R:TGGGCTTTAAGTCCCGATG	XM_021073087
NF-κBp65	F:CGAGAGGAGCACGGATACCA<br>R:GCCCCGTGTAGCCATTGA	NM_001114281
GSK-3β	F:CGAGACACACCTGCACTCTT<br>R:CCGGCATTAGTATCTGAGGCT	NM_001128443
β-catenin	F:GAGCTGAGAGCGAGGGGAG<br>R:ACAGCCGCTTTTCTGTCTGG	NM_214367
CCND1	F:TTGAAGGCGAGGTTCCAGTC<br>R:GCTGGTTCTCTAGGTCAGCC	XM_021082686
DKK1	F:AGGGTGGCAACAAGTACCAA<br>R:GCAGGCTAGGCAGATTTGTG	NM_001145384
DKK2	F:GCCTCGCCTTTGATTGCTTC<br>R:CCCACAACCCCACTTGACTT	XM_003129269
FZD7	F:TGGTGAAGGTGCAGTGTTCT<br>R:TGTAGGGCGCTGTAGGATAGG	XM_013984388
AXIN2	F:GCTATGGATTTTGGGGCCG<br>R:GTCCCTCTCAGCAATCAGCG	XM_021066736
c-Myc	F:TCCACGCACCAGCACAATTA<br>R:ATTGTGTGTCCGCCTCTTGT	NM_001005154
β-actin	F:TGGAACGGTGAAGGTGACAG<br>R:CTTTTGGGAAGGCAGGGACT	XM_003124280
GAPDH	F:CGGAGTGAACGGATTTGGC<br>R:CACCCCATTTGATGTTGGCG	NM_001206359

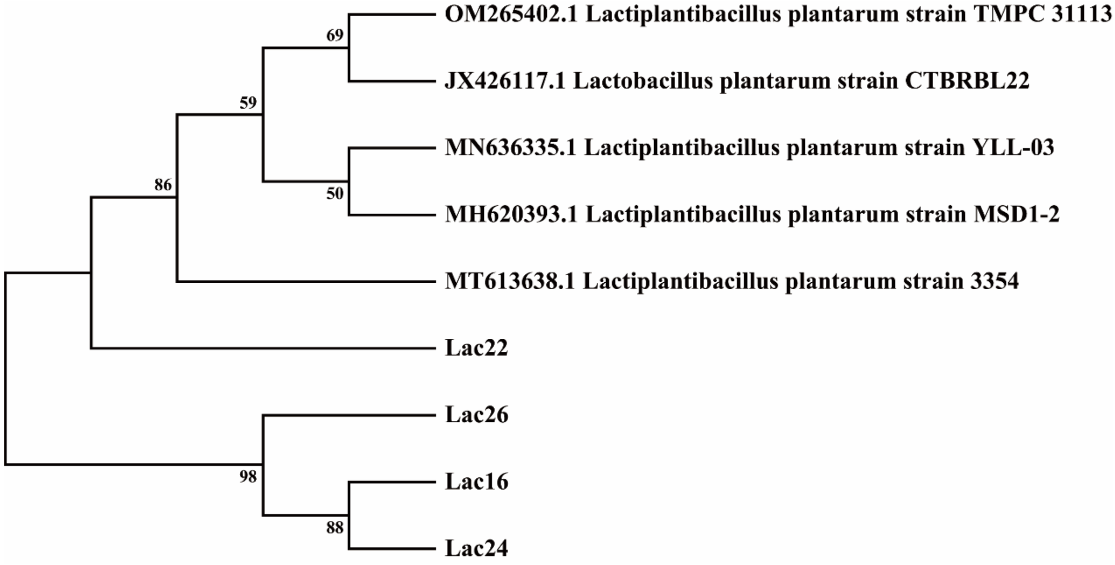

Figure S1 Phylogenetic tree of probiotics. The tree was constructed using software MEGA 10.1.8 by neighbor-joining method based on 16S rDNA gene sequences with 1000 replications in bootstrap test.
